# Current trends in canine dirofilariosis in Austria—do we face a pre-endemic status?

**DOI:** 10.1007/s00436-019-06576-4

**Published:** 2020-02-13

**Authors:** Karin Sonnberger, Georg G. Duscher, Hans-Peter Fuehrer, Michael Leschnik

**Affiliations:** 1 Tierklinik Sattledt Traunkeis Vet Clinic OG, Kirchdorfer Staße 7, 4642 Sattledt, Austria; 2 Institute of Parasitology, Department of Pathobiology, University of Veterinary Medicine Vienna, Veterinärplatz 1, 1210 Vienna, Austria; 3 Internal Medicine Small Animals Department for Companion Animals and Horses, University of Veterinary Medicine Vienna, Veterinärplatz 1, 1210 Vienna, Austria

**Keywords:** Canine dirofilariosis, *Dirofilaria repens*, *Dirofilaria immitis*, Zoonosis

## Abstract

A retrospective study based on cases of canine dirofilariosis presented to the University of Veterinary Medicine, Vienna or diagnosed by private practitioners throughout Austria, from 1998 to 2018 was conducted to investigate the long-term development and current state of canine dirofilarial infections in Austria. Included in this study were 146 dogs which were tested positive for *D. immitis* and/or *D. repens.* The most commonly used diagnostic methods and the probable geographical origins of the infections were evaluated and the treatment protocols applied were compared with each other and with the literature. The results show that most infections were found due to screening for common travel infections using antigen-ELISA or PCR-testing, or by the incidental finding of microfilariae. Remarkably, only 24.3% of all cases presented showed clinical signs indicating canine dirofilariosis. Regarding the origin and travel history of the dogs, thirteen different countries could be identified. The three treatment protocols used showed a similar outcome after 8 months of treatment and minor side effects, which is consistent with the literature. An alarming increase in reported infections with both *D. immitis* and *D. repens* in Austria was noted since 2014. The number of documented cases had almost tripled by 2018, raising severe concerns about the threat of it becoming endemic in Austria. Therefore, the existing recommendations in current guidelines regarding canine dirofilariosis should be widely publicised and more strictly enforced. Prophylactic measures for dogs travelling abroad and diagnostic and therapeutic strategies for dogs imported from endemic countries should be obligatorily established throughout Europe, to reduce the risk of further spread of canine filarial infections to non-endemic regions.

## Introduction

Dirofilariosis is a parasitic vector-borne infection, caused by different filarioid parasites of the genus *Dirofilaria* and can be considered an emerging disease in Europe (Morchón et al. 2012). Increasing numbers of dogs with a travel history to endemic regions, as well as dogs imported from endemic countries, may serve as carriers for both *D. repens* and *D. immitis* and introduce these parasites to non-endemic countries (Leschnik et al. 2008; Genchi et al. 2009).

Several mosquito species known to be competent vectors for *D. immitis* and *D. repens* are present in Austria (Fuehrer et al. 2016). In 2012, the first autochthonous findings of *D. repens* in mosquitoes were made in eastern Austria (Silbermayr et al. 2014). As climate change proceeds, more vectors for *D. immitis* may show increasing occurrence in Europe, which could possibly enable autochthonous transmission of dirofilariosis in previously unaffected countries (Leschnik et al. 2008; Genchi et al. 2009; Morchón et al. 2012; Kronefeld et al. 2014).

Up to 2014, Fuehrer et al. (2016) reported 37 infections with *D. repens* and 25 infections with *D. immitis* in dogs in Austria. Seven cases of cutaneous dirofilariosis were discussed as autochthonous infections; no wildlife hosts were detected in Austria. As *D. repens* and *D. immitis* both cause zoonotic infections and affect humans, showing a cutaneous, ocular or pulmonary manifestation, it is particularly important to prevent further spread across Europe. This concerns not only veterinary medicine but also public health. Since 1978, 33 cases of human dirofilariosis have been reported in Austria caused by *D. repens* (30 cases) and *D. immitis* (3 cases). *D. repens* infections manifested subcutaneous or ocular lesions, while *D. immitis* caused pulmonary symptoms in infected humans. One of the *D. repens* infections is suspected to be autochthonous as the patient had no travel history to endemic regions (Auer and Susani 2008).

In these circumstances, it is increasingly important to improve the communication and establishment of existing guidelines for diagnostic, therapeutic and prophylactic measures for dirofilarial infections in dogs, as has already been outlined by the American Heartworm Society (AHS), the European Society for Dirofilariosis and Angiostrongylosis (ESDA) and the European Scientific Counsel Companion Animal Parasites (ESCCAP). These guidelines, published by the above mentioned organisations, are based on scientific peer-reviewed publications and international scientific opinion leaders. They should be consistently applied in Austria and other non-endemic countries for dogs travelling to endemic regions with their owners and for potentially infected dogs imported from endemic areas, for example, by animal welfare organisations or through legal and illegal pet trade.

Canine subcutaneous dirofilariosis is most commonly an asymptomatic course, but it can also result in a variety of non-specific dermal alterations (Genchi et al. 2010; Petry et al. 2015). Possible cutaneous changes are nodules, pruritus, thin and fragile skin and ocular conjunctivitis (Hargis et al. 2002; Bourdeau and Roussel 2010; Albanese et al. 2013). Typically, there are no inflammatory reactions or capsules located around the parasite, which actively moves under the connective tissue layers (Genchi and Kramer 2017). In severe infections, allergic reactions associated with microfilaria sensitisation and *Wolbachia*-mediated inflammatory reactions have been reported (Rocconi et al. 2012). Occasionally, *D. repens* may be found in the pelvic cavity and mesentery (Mircean et al. 2017).

The clinical course of canine heartworm disease is generally divided into three stages. The first stage is an infection without any or with very mild symptoms, such as exercise-induced dyspnea, occasional coughing and mild apathy. In the second stage of infection, the animals usually show a chronic cough, dyspnea, poor performance and stress intolerance, as well as a dull coat, weight loss and mild anaemia. Frequent vomiting can also occur at this stage (Boreham and Atwell 1988; Polizopoulou et al. 2000). The third stage typically follows a longer course of the disease with a large worm burden. Affected dogs present tachycardia, tachypnoea and syncopes in addition to the symptoms seen in stage two (Boreham and Atwell 1988; Polizopoulou et al. 2000).

There are many different treatment protocols against micro- or macrofilariae, as well as protocols affecting all stages of *D. immitis*. These protocols differ in their duration, the pharmaceuticals used and the frequency of administration. As a consequence, they differ in the time, effort and costs involved and show a different risk potential for the patient. Slow kill protocols using monthly ivermectin-injections over a period of 24 months or topical application of imidacloprid and moxidectin at 30-day intervals for 10 months are in sharp contrast to short protocols, using only two melarsomine-dihydrochloride-injections 24 h apart (Venco et al. 2004; Bowman and Atkins 2009; Savadelis et al. 2017). Possible side effects during adulticidal therapy are rare and include anaphylactic shock or thromboembolism in the pulmonary arteries (Bowman and Atkins 2009). To avoid the development of such thromboembolisms, a strict limitation of movement during and several weeks after therapy is recommended (American Heartworm Society 2018). Using microfilaricidal therapy, side effects such as lethargy, vomiting, tachypnea or vasodilatation and (in rare cases) anaphylactic shock can be seen with high parasite burdens, due to the sudden death of a large number of microfilariae. In general, however, microfilaricidal treatments are well tolerated (Bowman and Atkins 2009; Deplazes et al. 2013). Surgical therapy might be considered in dogs showing severe clinical symptoms or vena cava syndrome. Adult worms are removed via the jugular vein using alligator forceps (Schnieder 2006). It is important to ensure complete and intact removal of the parasites in order to avoid an anaphylactic reaction to any remaining material (Deplazes et al. 2013). Ideally, treatment against *D. immitis* should affect the juvenile as well as adult stages and is used in combination or after pretreatment with doxycycline against the bacterial endosymbiont *Wolbachia* spp. (Deplazes et al. 2013; ESDA 2017; American Heartworm Society 2018). For the best practicability and animal owner acceptance, it should also be as simple, inexpensive and safe as possible.

In contrast, there is currently only one approved substance for the treatment of *D. repens* in dogs—moxidectin. However, since most infections cause no severe clinical symptoms, therapy is not necessarily required. In some cases, surgical removal of parasitic nodes is possible. Macrocyclic lactones should be used in infected dogs to reduce the microfilarial burden and the risk of transmission to other dogs or humans by infective vectors (ESCCAP 2012).

The aim of this study was to evaluate the long-term trend and current state of canine dirofilariosis in Austria, to present data on the estimated origins of these infections, to investigate why and how a diagnosis or suspected diagnosis was made and to compare the treatment protocols applied against *D. immitis*.

## Materials and methods

Cases of canine dirofilariosis presented to the University of Veterinary Medicine, Vienna (68 dogs) or diagnosed by private practitioners in Austria and reported to the University for therapeutic advice (78 dogs) from 1998 to 2018 were retrospectively analysed. Data were collected regarding the age, sex and breed of dogs, their country of origin and travel history and the presence of clinical signs of dirofilariosis. The diagnostic methods applied and their results, as well as the therapy protocols used and their outcome, were assessed.

## Results

### General findings

Included in this study were 146 dogs living in Austria which were tested positive for *D. immitis* and/or *D. repens* from 1998 to 2018. Most dogs were situated in central or eastern Austria. Ninety-nine dogs were diagnosed with *D. immitis*, 37 dogs with *D. repens* and ten dogs had a co-infection with both nematodes.

Sixty-three of the dogs were female and 83 male. Twenty-eight dog breeds were represented, however, 63% of the dogs were cross-bred. The average age of the dogs was 4.8 ± 2.5 years (mean ± SD), ranging from 1 to 13 years. The clinical staging of all *D. immitis* infected dogs (*n* = 109) resulted in 86 dogs without clinical or with mild signs (stage one), fifteen dogs showing clinical signs consistent with stage two and eight dogs in stage three.

### Country of origin and travel history

**Fig. 1:**
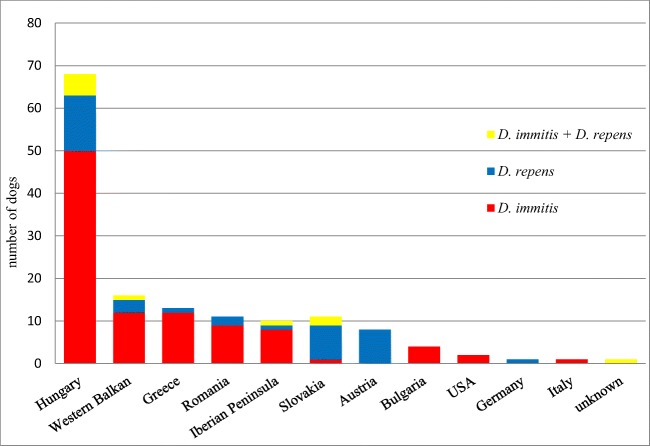
Travel history and origin of infected dogs

To determine where the presented dogs were most likely to have become infected with dirofilariosis, their origin and history of travelling were documented. Fourteen different countries could be identified. The dogs tested positive for *D. repens* had travel histories to or originated from Hungary, Greece, the western Balkans (Croatia, Serbia and Slovenia), the Iberian peninsula (Spain or Portugal), Romania, Slovakia or Germany. Eight *D. repens*-positive dogs had no reported travel history and originated from Austria. The dogs tested positive for *D. immitis* originated from Hungary, Greece, the western Balkans, the Iberian peninsula, Romania, USA or Bulgaria. An overview of travel histories and origins is shown in Fig. 1.

### Incidence of dirofilarial infections in Austria

**Fig. 2:**
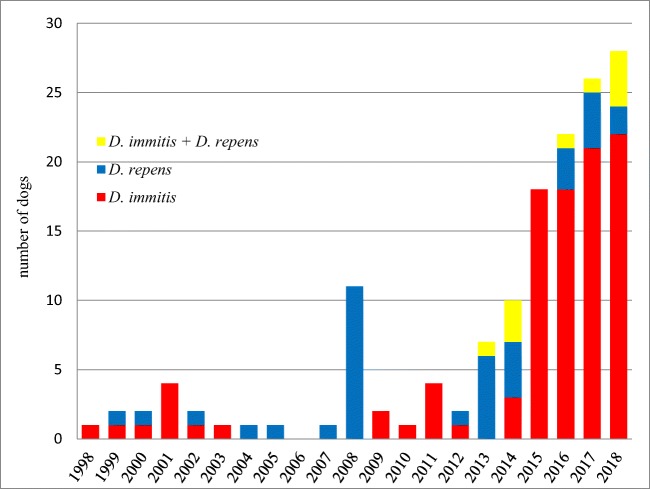
Trend of *D. immitis* and *D. repens* infections documented in dogs in Austria from 1990 to 2018

Before 2008, a maximum of four canine dirofilariosis cases per year was documented at the University of Veterinary Medicine, Vienna. In 2008, seven blood samples of asymptomatic dogs from eastern Austria were tested positive for *D. repens* in a scientific study (Duscher et al. 2009). Since 2014, there has been an explicit increase in reported infections with *D. immitis* and *D. repens,* with the highest number of 28 cases documented in 2018. The trend of canine dirofilariosis in Austria is shown in Fig. 2.

### Reasons for testing and diagnosing canine dirofilariosis

**Fig. 3:**
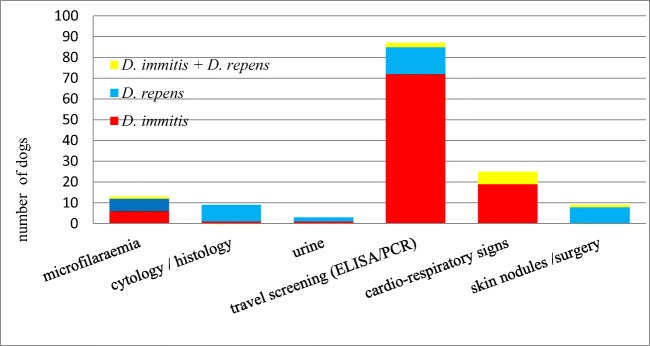
Reasons for diagnosing or suspecting dirofilariosis in the presented cases

Most *Dirofilaria* spp*.* infections were diagnosed using a screening test for common travel infections (59.6% in total; 72.7% of the *D. immitis* infections, 35.1% of the *D. repens* infections and 20% of co-infections) such as antigen-ELISA (71.23%) or PCR-testing (36.30%). A total of 17.2% of the dogs (8.1% of the *D. immitis* infections, 43.2% of the *D. repens* infections and 10% of the double infections) were diagnosed by an incidental finding of microfilariae in the blood smear (*n* = 13), mass cytology or histology (*n* = 9) or urine (*n* = 3). Adult *D. repens* were found within the skin nodules of nine dogs. Twenty-three dogs were screened for heartworm disease because of cardiorespiratory symptoms and cardio-ultrasonographical findings. Sixty percent of the co-infected dogs were tested for dirofilariosis because of cardiorespiratory signs. For a correct staging of the cardiopulmonary dirofilariosis, thoracic X-rays and heart ultrasound should be performed; however, due to lack of clinical symptoms, most of the dogs did not undergo these examinations. The different reasons for testing and diagnosing canine dirofilariosis are displayed in Fig. 3.

### Treatment and follow up

Out of 109 dogs diagnosed with *D. immitis*, 86 received heartworm treatment and 49 treated dogs were monitored over a period for a minimum of 6 months. The 49 dogs were tested for *D. immitis*-antigen 3 months or later after treatment. Fifty-six dogs with clinical signs consistent with first or second stage heartworm disease were treated with a two-injection protocol with melarsomine dihydrochloride including pre-treatment with doxycycline and a macrocyclic lactone mentioned by Hinney and Leschnik (2015). Thirty dogs were treated according to the protocol recommended by the American Heartworm Society (AHS) (three injections of melarsomine after pre-treatment with doxycycline and a macrocyclic lactone) or, if requested by the owner, by a slow kill protocol. Dogs infected with *D. repens* were treated with macrocyclic lactones over a period of 6 months.

In dogs treated with a protocol using melarsomine dihydrochloride, 91.11% showed negative test results within 8 months after treatment. Documented side-effects, such as local pain reactions at the injection site, were common, but mild and self-limiting within 24 h. One dog died of acute lung failure during an AHS-protocol treatment.

Four owners opted for a slow kill protocol and the monthly moxidectin application resulted in a negative antigen test result in three dogs respectively after six, nine and 10 months, respectively. One dog remained positive in the antigen test after 10 months of treatment.

Three dogs died or were euthanized for reasons not related to dirofilariosis. No information about the therapy methods applied was available for six dogs. Ten dogs with cardiopulmonary dirofilariosis and eleven dogs with cutaneous dirofilariosis received no therapy at the owners’ request, even though treatment was urgently recommended. The remaining dogs were lost during follow-up or therapy started only within the last 6 months.

## Discussion and conclusion

Due to their increasing prevalence, infections with *Dirofilaria immitis* and *Dirofilaria repens* have become a rapidly growing concern in Central Europe in recent years.

*D. immitis* is predominantly found in tropical to subtropical climates but can be seen progressively emerging in countries with rather mild climates adjacent to these regions. The parasite is found in many parts of USA, in multiple African and South Asian countries, as well as in Australia and Europe (Boreham and Atwell 1988; Deplazes et al. 2013). The main distribution areas in Europe are located in southern countries, like France, Greece, Italy, Portugal and Spain. The largest endemic region in Europe is the Padan Plain in Northern Italy, where canine dirofilariosis can be found with a prevalence of 50–80% (Genchi et al. 2005). *D. repens* has spread even faster to Northern Europe than *D. immitis*, which could be due to the more frequent subclinical infections and therefore undiagnosed dogs can serve as reservoirs and carriers. It also appears that *D. immitis* is establishing itself more slowly in areas where *D. repens* is already present (Genchi et al. 2005; Capelli et al. 2018).

According to their owners, eight of the 146 dogs included in this study never left Austria. Most of the infected dogs had been imported from neighbouring countries and all of them were classified adults at the time of crossing the borders. Regarding the origin and travel history of the dogs, fourteen different countries could be identified. Most dogs infected with *D. immitis* came from (or had a travel history to) Hungary and Greece, both are endemic countries for dirofilariosis disease. These results are slightly different from a study in Germany, where most cases of *D. immitis* were imported from Greece and Spain and only one case from Hungary (Pantchev et al. 2011). In Greece, the prevalence of *D. immitis* was 4.1% out of 750 dogs examined in 2016 and the highest prevalence could be found in northern Greece, where most of the dogs in the present study with a travel history to Greece had travelled (Diakou et al. 2016). While *D. repens* is known to occur in dogs throughout Hungary, canine cardiopulmonary dirofilariosis has so far been documented in only a few individual cases (Genchi et al. 2005; Fok 2007; Jacsó et al. 2009; Tolnai et al. 2014). It is noteworthy that not only has the number of reported cases of heartworm disease in Hungarian dogs risen since 2012, but also the geographic distribution of *D. immitis* has disseminated from a few cases in the south of the country to the whole of eastern and central Hungary (Farkas et al. 2014). Most of the dogs originating from Hungary in the present study were from the region of the Great Hungarian Plain, which has been considered an endemic region since 2011 (Bacsadi et al. 2016).

In the present study, countries most commonly mentioned in the history of dogs infected with *D. repens* were Hungary, Slovakia and eastern Austria, which are again endemic regions for this infection (Tolnai et al. 2014; Fuehrer et al. 2016; Miterpáková et al. 2016). These results are only partly consistent with a German study from 2011, where most infections with *D. repens* were imported from Hungary, Greece and Italy (Pantchev et al. 2011).All countries mentioned are neighbouring countries of Austria or frequently visited travel destinations and are strongly represented by animal welfare organisations bringing shelter and stray dogs to Austria. The situation in Austria is comparable to a study from Germany, stating an increasing number of dogs imported from abroad or having travelled to other European and non-European states (Glaser and Gothe 1998). The spread of dirofilarial infections can be seen in many other European countries. Two studies from Bulgaria found that out of 80 dogs, 15% were positive for infection with *D. immitis* and 18% for infection with *D. repens* in 2016. In 2017, 34.33% of 367 dogs were positive for *D. immitis* and 5.45% positive for *D. repens* (Radev et al. 2016; Iliev et al. 2017). In Romania, *D. immitis* was found in three and *D. repens* in four different wild carnivore species in 2017, indicating a considerable reservoir in wild carnivores (Ionică et al. 2017a).

One problem of successfully monitoring the introduction of *D. immitis* and *D. repens* from endemic countries is that canine dirofilariosis often remains asymptomatic in its course and many affected dogs show no symptoms even after the long incubation period of at least five to 6 months (Schnieder 2006). Screening dogs from endemic countries only once for dirofilariosis before they are brought to non-endemic countries is therefore insufficient and tests should be repeated around 6 months after entering the non-endemic region, even if the dogs still appear asymptomatic.

In the years before 2014, only a few infections with canine dirofilariosis were reported in Austria. The annual number of reported cases increased from 111 in 2013 to 10 and 28 in the years 2014 and 2018 (Fig. 2). This might be due to the increasing number of dogs with a travel history to endemic regions and dogs imported from endemic countries, but also due to rising awareness of travel infections affecting dogs and, therefore, an increased and more adequate screening for associated diseases (Leschnik et al. 2008; Genchi et al. 2014). An issue which the current study also raises is the considerable increase in imports of adult dogs, with the consequence that more dogs that have been exposed to the pathogen for a potentially longer period of time are entering the country. Nevertheless, the current study has shown a significant, rapidly progressing and, therefore, non-negligible increase in a serious infectious disease and zoonosis. With several mosquito species in Austria potentially able to transmit *Dirofilaria* spp. and the expected increase of vectors in Central and Northern European countries due to advancing climate change, the increasing prevalence of dirofilariosis in dogs is a serious risk. The endemisation of dirofilariosis in Austria might only be a matter of time and is probably already concluded for *D. repens* (Morchón et al. 2012; Fuehrer et al. 2016; Capelli et al. 2018). Under these circumstances, the observed increase in canine heartworm infections in Austria could be considered a pre-endemic status.

In the present study, a total of 17.2% of the dogs were diagnosed only by accidental findings, like microfilaraemia, microfilariae in the urine or in cytological or histological preparations. Only 23.3% of all the dogs presented showed clinical signs indicating canine dirofilariosis, which is consistent with preceding studies, showing a long, symptom-free incubation period and many altogether asymptomatic cases of canine dirofilariosis (Schnieder 2006; Bowman and Atkins 2009). It is noteworthy that co-infected dogs showed more clinical signs (60.0%) than dogs infected with only *D. immitis* (16.84) or *D. repens* (23.33). Co-infections seem to be common in areas where both parasites are endemic (Ionică et al. 2017b). A direct interaction between both parasites is assumed, which affects the release of microfilariae to the blood as well as the host’s immune response (Genchi et al. 1995). One reason for the high percentage of coincidentally diagnosed infections could be a low incidence of this disease on the differential diagnosis lists of Austrian veterinarians. This could be due to a significantly lower awareness among veterinarians of *Dirofilaria*-related infections in non-endemic countries compared with their colleagues in endemic regions (Genchi et al. 2014).

Most *D. immitis* infections (67.9%) were diagnosed using a screening test for common travel infections and most *D. repens* infections (36.23%) were an incidental finding. Screening for travel infections was conducted using antigen-ELISA (71.23%) or PCR-testing (36.3%). The frequent use of in-house antigen-ELISA tests is likely to be due to their direct availability in clinical practice and the comparatively high sensitivity and specificity of the results obtained within minutes, even in dogs with a low worm burden (Courtney and Zeng 2001; Atkins 2003; Lee et al. 2011). A possible explanation for the difference in diagnostic methods used between the two filarial species would be the lack of a rapid screening system designed specifically for *D. repens*, as there exists for *D. immitis* and other common travel diseases (Capelli et al. 2018). Yet, even a specific screening system may be susceptible to cross-reactions, as well as false-negative or false-positive results, as shown in previous studies (Venco et al. 2017). Another factor for inconsistency is the pre-treatment with macrocyclic lactones, which can lead to the formation of antigen–antibody-complexes masking the antigen, which could then no longer be detected by commercial test kits (Drake et al. 2015). The use of heat-treated serum for in-house antigen tests can provide a solution to this problem and therefore has been practised in the context of this study since 2015 (Velasquez et al. 2014; DiGangi et al. 2017). However, a recent study found that heat treatment of serum samples can lead to false-positive results in dogs that have been successfully treated against macrofilariae but in whose blood non-degraded antigen is still present and that the pre-treatment with heat can also increase the susceptibility to cross-reactions (Savadelis et al. 2018).

Among the 131 dogs screened for microfilariae in the blood by microscopic blood smear examination or PCR, 66.4% showed microfilaraemia, which makes screening for microfilariae a helpful diagnostic tool. Nevertheless, in asymptomatic infections, microfilaraemia is seen in only 50% of the tested blood samples. Additionally, long prepatency, infections with only male or female macrofilariae or, as mentioned above, pre-treatment with macrocyclic lactones can lead to false-negative results (Deplazes et al. 2013; Drake et al. 2015). Therefore, a thorough screening for common travel infections in dogs with a history of travelling to endemic countries or having been born abroad is strictly recommended.

Classic adulticide treatment against *D. immitis* includes two injections of melarsomine dihydrochloride 24 h apart. Compared with the current American Heartworm Society treatment protocol, the classic regime leads more frequently to pulmonary thromboembolism, thus resulting in higher complication rates during the treatment period (Carreton et al. 2014). Possible complications, as well as the time and costs involved, can lead to pet owners opting for a slow kill protocol, demanding alternative therapeutic options or even refraining from any form of therapy (Ku 2017). It may sometimes be difficult for practitioners to accurately implement the therapy suggestions of the current guidelines in practice. However, in this threatening pre-endemic status, every effort should be made to remove each individual dog from the transmission cycle and, therefore, each infected dog must undergo appropriate therapy. For this reason, a melarsomine-protocol consisting of two injections (Hinney and Leschnik 2015) is used at the University for Veterinary Medicine, Vienna in dogs with heartworm disease stages one or two, showing a similar outcome compared with the literature (McTier et al. 1994; Maxwell et al. 2014; Bowman and Atkins 2009). In more recent studies, the assumption was made that starting therapy with macrocyclic lactones and melarsomine dihydrochloride shortly after diagnosis achieves the highest therapeutic success and prevents the growth of a large worm burden. A waiting period of several months from diagnosis to therapy is therefore not recommended, unless in severely symptomatic patients (Bowman and Drake 2017). Regarding dogs imported from endemic areas, it is more difficult to consider the susceptibility gap, which is defined as the period when adult heartworm disease has been diagnosed, but some stages of *D. immitis* are not susceptible to treatment with macrocyclic lactones or melarsomine dihydrochloride (Bowman and Drake 2017), as many dogs are presented several months after their entry, that is a long time after exposure to the parasite. Irrespective of the therapy protocol used, administration of macrocyclic lactones and doxycycline one to 2 weeks prior to the first melarsomine injection should be considered in order to minimize side effects induced by the death of macrofilariae (McCall 2005; McCall et al. 2014).

In conclusion, canine dirofilariosis in Austria has shown an alarming increase in the past few years and appropriate interventions are needed to counter this development which can be considered exemplary for the endemisation of a vector-borne parasitosis and zoonosis in a hitherto non-endemic country. Awareness of practitioners and owners about the potential risk of infection with dirofilariosis while travelling to endemic regions or importing dogs from endemic countries should be raised. The zoonotic potential of dirofilariosis and the severity of this disease in some human cases should be communicated more strongly, as the long incubation period and asymptomatic courses may lead to unnoticed cases of dirofilarial infections in dogs who are living in close owner–pet-relationships. Thus, it is a non-negligible risk to animal and human health (Bazzocchi et al. 2008; Kartashev et al. 2017). Prophylactic measures for dogs travelling abroad and diagnostic and therapeutic strategies for dogs imported from endemic countries, as have already been outlined by the American Heartworm Society (AHS), the European Society for Dirofilariosis and Angiostrongylosis (ESDA) and the European Scientific Counsel Companion Animal Parasites (ESCCAP), should be obligatorily established throughout Europe, to reduce the risk of further spread of canine filarial infections to non-endemic regions.
